# CRP Is an Activator of *Yersinia pestis* Biofilm Formation that Operates via a Mechanism Involving *gmhA* and *waaAE-coaD*

**DOI:** 10.3389/fmicb.2016.00295

**Published:** 2016-03-08

**Authors:** Lei Liu, Haihong Fang, Huiying Yang, Yiquan Zhang, Yanping Han, Dongsheng Zhou, Ruifu Yang

**Affiliations:** ^1^State Key Laboratory of Pathogen and Biosecurity, Beijing Institute of Microbiology and EpidemiologyBeijing, China; ^2^Department of Biochemistry, School of Medicine, Jiangsu UniversityZhenjiang, China

**Keywords:** *Yersinia pestis*, CRP, *gmhA*, *waaAE-coaD*, *phoPQ*-YPO1632, biofilm

## Abstract

*gmhA* encodes a phosphoheptose isomerase that catalyzes the biosynthesis of heptose, a conserved component of lipopolysaccharide (LPS). GmhA plays an important role in *Yersinia pestis* biofilm blockage in the flea gut. *waaA*, *waaE*, and *coaD* constitute a three-gene operon *waaAE*-*coaD* in *Y. pestis*. *waaA* encodes a transferase that is responsible for binding lipid-A to the core oligosaccharide of LPS. WaaA is a key determinant in *Y. pestis* biofilm formation, and the *waaA* expression is positively regulated by the two-component regulatory system PhoP/PhoQ. WaaE is involved in LPS modification and is necessary for *Y. pestis* biofilm production. In this study, the biofilm-related phenotypic assays indicate that the global regulator CRP stimulates *Y. pestis* biofilm formation *in vitro* and on nematodes, while it has no regulatory effect on the biosynthesis of the biofilm-signaling molecular 3′,5′-cyclic diguanosine monophosphate. Further gene regulation experiments disclose that CRP does not regulate the *hms* genes at the transcriptional level but directly promotes the *gmhA* transcription and indirectly activates the *waaAE-coaD* transcription through directly acting on *phoPQ*-YPO1632. Thus, it is speculated that CRP-mediated carbon catabolite regulation of *Y. pestis* biofilm formation depends on the CRP-dependent carbon source metabolic pathways of the biosynthesis, modification, and transportation of biofilm exopolysaccharide.

## Introduction

*Yersinia pestis*, the causative agent of plague, is transmitted primarily by fleas and has been responsible for three plague pandemics in human history (Perry and Fetherston, 1997). Biofilms formed by *Y. pestis* can attach to and block the proventriculus of a flea, which promotes the flea-borne transmission of this pathogen (Zhou and Yang, 2011; Chouikha and Hinnebusch, 2012). HmsHFRS is responsible for the biosynthesis and translocation of poly-β-1,6-*N*-acetylglucosamine exopolysaccharide (EPS), a primary dry component of the *Y. pestis* biofilm matrix (Bobrov et al., 2008). In *Y. pestis*, HmsT and HmsD (*hmsD* is located in the *hmsCDE* operon) are the two sole diguanylate cyclases that catalyze the biosynthesis of 3′,5′-cyclic diguanosine monophosphate (c-di-GMP), a second messenger stimulating biofilm EPS production (Bobrov et al., 2011; Sun et al., 2011). Conversely, c-di-GMP is degraded by the sole phosphodiesterase HmsP in *Y. pestis* (Kirillina et al., 2004; Bobrov et al., 2005).

Lipopolysaccharide (LPS) is an integral component of the outer membrane of Gram-negative bacteria. Generally, it is composed of three domains: lipid-A, core oligosaccharide, and O-specific antigen or O side chain. However, the LPS from *Y. pestis* contains only lipid-A bound to the core oligosaccharide by 3-deoxy-D-manno-octulosonic acid (Kdo) (Prior et al., 2001). The *gmhA* gene encodes a phosphoheptose isomerase that is required for heptose biosynthesis, a conserved component of the core oligosaccharide, and the deletion of *gmhA* in *Y. pestis* leads to inadequate biofilm production for flea blockage (Darby et al., 2005). In *Y. pestis*, *waaA*, *waaE*, and *coaD* constitute a three-gene operon *waaAE*-*coaD*. The *waaA* gene encodes a Kdo transferase involved in the attachment of lipid-A to the core oligosaccharide, and *waaA* mutants show a severely biofilm-defective phenotype of *Y. pestis* (Tan and Darby, 2005). The *waaE* gene encodes a protein that is required for adding a substitution on the inner core heptose, and the *waaE* deletion leads to 40% attenuation of biofilm production of *Y. pestis* (Izquierdo et al., 2002).

PhoP and PhoQ constitute a typical two-component regulatory system (Groisman, 2001). The PhoPQ system is necessary for the intracellular survival of *Y. pestis* in phagocytes during the early stages of infection because the PhoP-regulated genes promote the resistance to antimicrobial peptides and the adaptation to low-Mg^2+^ conditions (Oyston et al., 2000; Grabenstein et al., 2006). However, the deletion of *phoP* in *Y. pestis* contributes little to plague pathogenesis in mice (Bozue et al., 2011). In addition, PhoPQ is induced by low pH in the flea gut, and controls physiological adaptation to acid and other stresses encountered during infection of the fleas; it simultaneously stimulates the formation of flea-borne infectious *Y. pestis* biofilms (Rebeil et al., 2013; Vadyvaloo et al., 2015). Nevertheless, PhoP-mediated regulation of *Y. pestis* biofilm formation relies on its modulation of the *waaAE-coaD* operon rather than the *hms* genes (Liu et al., 2014).

Cyclic AMP receptor protein (CRP) has been characterized as a virulence-associated regulator in many pathogens (Skorupski and Taylor, 1997; Petersen and Young, 2002; Rickman et al., 2005; Zhan et al., 2008; Oh et al., 2009). CRP controls the expression of multiple bactieral virulence genes and responds to environmental changes by sensing the availability of carbons (Busby and Ebright, 1999). CRP can only be activated by binding a small-molecule inducer cyclic AMP (cAMP), and the cAMP-CRP complex usually serves as a DNA-binding transcription factor by directly binding to the promoter regions of its target genes to activate or repress their transcription (Busby and Ebright, 1999). The cAMP-CRP complex modulates more than 6% of *Y. pestis* genes that are involved in a large variety of functions (Zhan et al., 2008). CRP is required for the development of bubonic and pneumonic plague, which may rely on CRP-dependent expression of *pla* and that of the *sycO-ypkA-yopJ* operon of the Yop-Ysc type III secretion system in *Y. pestis* (Kim et al., 2007; Liu et al., 2009; Zhan et al., 2009). Meanwhile, *crp* expression is positively regulated by PhoP at the transcriptional level and by Hfq (a major posttranscriptional regulator that controls biofilm formation and virulence in *Y. pestis*) at the posttranscriptional level (Zhang et al., 2013a; Lathem et al., 2014), indicating the significant role of CRP in the virulence gene regulation of *Y. pestis*. CRP or its homologous regulators control biofilm production in various pathogens such as *Listeria monocytogenes* (Salazar et al., 2013), *Xanthomonas campestris* pathovar *campestris* (Lu et al., 2012), *Aggregatibacter actinomycetemcomitans* (Torres-Escobar et al., 2013), *Vibrio cholera* (Fong and Yildiz, 2008), and *Serratia marcescens* (Kalivoda et al., 2008). The loss of CRP in *Y. pestis* results in a significant reduction of biofilm production, and the carbon storage regulator CsrA can enhance CRP-mediated regulation of *Y. pestis* biofilm formation (Willias et al., 2015). However, the crucial CRP-dependent factors that contribute to this alteration of biofilm formation are still unknown.

In the present work, the biofilm-related phenotypic assays showed that deletion of *crp* in a *Y. pestis Microtus* strain led to a dramatic reduction in biofilm production, but had no effect on the c-di-GMP biosynthesis. The subsequent gene regulation experiments indicated that CRP directly activated the *gmhA* transcription and meanwhile indirectly stimulated the *waaA* transcription through directly acting on *phoPQ*, while CRP had no regulatory effect on the *hms* genes at the transcriptional level.

## Materials and Methods

### Ethics Statement

All the animal experiments were carried out in accordance with the protocols approved by the Ethics Committee in the Beijing Institute of Microbiology and Epidemiology.

### Bacterial Strains

The wild-type *Yersinia pestis Microtus* strain 201 (WT) is avirulent to humans but highly virulent to mice (Zhou et al., 2004). The non-polar *crp* mutant (Δ*crp*) and its complemented mutant strain (*C-crp*) have been described previously (Zhan et al., 2008). Given the previous observation that deletion of *hmsS* led to a biofilm-defective phenotype in *Y. pestis* (Forman et al., 2006), the *hmsS* mutant Δ*hmsS* was used as a reference biofilm-defective strain in this work. All of the primers designed in this study are listed in Supplementary Table S1.

### Bacterial Growth and RNA Isolation

Overnight cell cultures in Luria-Bertani (LB) broth with an optical density (OD_620_) of about 1.0 were diluted 1:20 into 18 ml of fresh LB broth for further cultivation at 26°C with shaking at 230 rpm to reach middle stationary phase (an OD_620_ of 1.0 to 1.2), then cells were harvested for gene regulation or phenotypic assays. Immediately before harvesting of bacterial cells for RNA isolation, a double-volume of RNAprotect reagent (Qiagen) was added to one-volume of cell culture, and total RNA was extracted using TRIzol Reagent (Invitrogen). RNA quality was monitored by agarose gel electrophoresis, and RNA quantity was determined by spectrophotometry.

### Primer Extension Assay

As described in our previous studies (Sun et al., 2012; Zhang et al., 2013a,b) a 5′-^32^P-labeled oligonucleotide primer complementary to a portion of the RNA transcript of each indicated gene was employed to synthesize cDNAs from total RNA templates using the Promega Primer Extension System. If different *Y. pestis* strains were involved in a single experiment, equal amounts of total RNA were used as starting materials. Sequence ladders were prepared with the same 5′-^32^P-labeled primers using the AccuPower and Top DNA Sequencing Kit (Bioneer). Radioactive species were detected by autoradiography. The 5′-terminus of the RNA transcript (i.e., transcription start) of each target gene was mapped according to the size of primer extension products, while the relative mRNA levels were determined by the intensities of the primer extension products.

### Quantitative RT-PCR

Gene-specific primers were designed to produce amplicons for target genes. Contaminating DNA in the RNA samples was removed using the Ambion DNA-free^TM^ Kit (Applied Biosystems). cDNAs were generated using 5 μg of RNA and 3 μg of random hexamer primers. Real-time PCR was performed using the LightCycler system (Roche) and the SYBR Green master mix (Zhan et al., 2008; Sun et al., 2014). Based on the standard curves of 16S rRNA expression, the relative mRNA level was determined by calculating the threshold cycle (ΔCt) of target genes via the classic ΔCt method. Negative controls used cDNA generated without reverse transcriptase as a template. Reactions containing primer pairs without template were also included as blank controls. The 16S rRNA gene was used as an internal control for normalization.

### LacZ Fusion and β-Galactosidase Assay

A promoter-proximal DNA region of each indicated gene was cloned into the low-copy-number transcriptional fusion vector pRW50 (Lodge et al., 1992) that harbors a promoterless *lacZ* reporter gene. *Y. pestis* strains transformed with the recombinant plasmid or the empty pRW50 (negative control) were cultivated to measure β-galactosidase activity in the cellular extract using the β-galactosidase Enzyme Assay System (Promega) (Sun et al., 2012; Zhang et al., 2013a,b).

### Purification of 6x His-Tagged CRP (His-CRP) Protein

The entire coding region of *crp* of strain 201 was directionally cloned into the *Bam*HI and *Hin*dIII sites of plasmid pET28a (Novagen) as described previously (Zhan et al., 2008). The recombinant plasmid encoding the His-CRP protein was transformed into *Escherichia coli* BL21 (DE3) cells (Novagen). Expression of His-CRP was induced by the addition of 1 mM isopropyl-beta-D-thiogalactoside. The overproduced proteins were purified under native conditions using a Ni-NTA agarose column (Qiagen). The purified, eluted protein was concentrated with the Amicon Ultra-15 (Millipore) to a final concentration of 0.8–1.0 mg/ml in storage buffer containing phosphate buffered saline (PBS, pH 9.5) plus 20% glycerol. The protein purity was verified by sodium dodecyl sulfate polyacrylamide gel electrophoresis with silver staining.

### Electrophoretic Mobility Shift Assay (EMSA)

For EMSA (Sun et al., 2012; Zhang et al., 2013a), promoter-proximal DNA regions were prepared by PCR amplification. EMSA was performed using the Gel Shift Assay Systems (Promega). The 5′ ends of DNA were labeled using [γ-^32^P] ATP and T4 polynucleotide kinase. DNA binding was performed in a 10 μl reaction volume containing binding buffer [100 μM MnCl_2_, 1 mM MgCl_2_, 0.5 mM DTT, 50 mM KCl, 10 mM Tris-HCl (pH 7.5), 0.05 mg/ml sheared salmon sperm DNA, 0.05 mg/ml BSA, and 4% glycerol], labeled DNA (1000 to 2000 c.p.m/μl), 1 mM cAMP, and increasing amounts of His-CRP. We included two control reactions: one contained the specific DNA competitor (unlabeled promoter DNA regions; cold probe), while the other was the non-specific protein competitor (rabbit anti-F1-protein polyclonal IgG antibody). After incubation at room temperature for 30 min, the products were loaded onto a native 4% (w/v) polyacrylamide gel and electrophoresed in 0.5x Tris-borate buffer containing 100 μM MnCl_2_ for 60 min at 150 V. Radioactive species were detected by autoradiography.

### DNase I Footprinting

For DNase I footprinting (Zhang et al., 2013a,b), the target promoter-proximal DNA regions with a single ^32^P-labeled end were incubated with increasing amounts of purified His-CRP, which was followed by partial digestion with RQ1 RNase-Free DNase I (Promega). The digested DNA samples were extracted with phenol/chloroform, precipitated with ethanol, and analyzed in a 8 M urea-6% polyacrylamide gel. Protected regions were identified by comparison with the sequence ladders. For sequencing, we used the Top DNA Sequencing Kit (BIONEER). The templates for sequencing were the same as the DNA fragments in the DNase I footprinting assay.

### Biofilm-Related Assays

Four different methods (Fang et al., 2013, 2015) were employed for biofilm-related assays: (i) crystal violet staining of the *in vitro* biofilm masses attached to the well walls when bacteria were grown in polystyrene microtiter plates; (ii) determination of the percentages of fourth-stage larvae and adults (L4/adult) of *Caenorhabditis elegans* (Darby et al., 2002; Fang et al., 2013) after the incubation of nematode eggs on *Y. pestis* lawns, which negatively reflected the bacterial ability to produce biofilms; (iii) observation of the rugose colony morphology of bacteria grown on LB agar plates, which positively reflected the bacterial ability to synthesize biofilm matrix biofilm EPS; and (iv) determination of intracellular c-di-GMP levels by a chromatography-coupled tandem mass spectrometry (HPLC-MS/MS) method.

### Experimental Replicates and Statistical Methods

For real-time RT-PCR, LacZ fusion, crystal violet staining of biofilms, and the determination of L4/adult nematodes or c-di-GMP, experiments were performed with at least three independent bacterial cultures/lawns, and values were expressed as the mean ± SD. The paired Student’s *t*-test was performed to determine significant differences; *P* < 0.01 was considered to indicate statistical significance. For primer extension, EMSA, DNase I footprinting, and colony morphology observation, representative data from at least two independent biological replicates are shown.

## Results

### CRP Enhances Biofilm Formation, But Does Not Influence c-di-GMP Production

**FIGURE 1:**
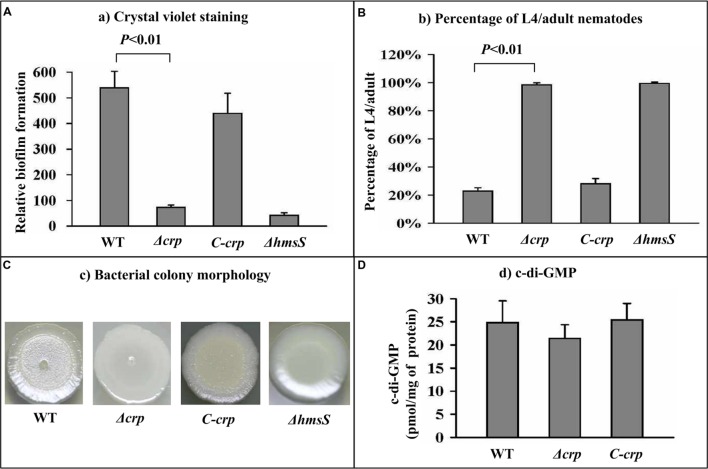
**Involvement of CRP in biofilm formation and c-di-GMP biosynthesis. (A)** Crystal violet staining. *Yersinia pestis* was grown in 24-well polystyrene dishes, and the bacterial biomass (*in vitro* biofilms) attached to well walls were stained with crystal violet to determine OD_570_ values. The planktonic cells were used to determine OD_620_ values. The relative capacity for biofilm formation of each strain was determined as follows: 100 × OD_570_/OD_620_. **(B)** *C. elegans* biofilms. After incubation of nematode eggs on lawns of the indicated *Y. pestis* strains, the developmental stages of nematodes on each lawn were scored to calculate the percentage of L4/adults. **(C)** Bacterial colony morphology. Aliquots of bacterial glycerol stocks were spotted onto LB plates, followed by incubation for one week. **(D)** Intracellular c-di-GMP concentration. The intracellular c-di-GMP concentrations were determined by a HPLC-MS/MS method, and the determining values were expressed as pmol/mg of bacterial protein.

Both the Δ*crp* and Δ*hmsS* mutant strains showed a dramatic reduction in biofilm crystal violet staining compared with the WT and the complemented mutant strain *C-crp*, indicating the significantly decreased production of *in vitro* biofilms due to the deletion of *crp* (**Figure 1A**). After incubation of nematode eggs on bacterial lawns of WT or *C-crp*, only a small portion (about 20%) of larvae grew and developed into L4/adult nematodes owing to abundant attachment of *Y. pestis* biofilms on nematode heads; while the value for the Δ*crp* and Δ*hmsS* mutants was nearly 100% (**Figure 1B**). When grown on LB agar plates, Δ*crp* developed much smoother colony morphology than WT that was comparable to *C-crp* (**Figure 1C**). This observation suggested that Δ*crp* produced much less biofilm EPS relative to WT. The intracellular levels of c-di-GMP were determined in the WT, Δ*crp*, and *C-crp* strains by a HPLC-MS/MS method. However, Δ*crp* showed no difference in the c-di-GMP production compared with WT and *C-crp* (**Figure 1D**). In summary, CRP was an activator of *Y. pestis* biofilm development, but had no effect on c-di-GMP production.

The first genes of the multi-gene operons *hmsHFRS*, *hmsCDE*, *phoPQ*-YPO1632, and *waaAE-coaD* as well as the individual genes *hmsT*, *hmsP*, and *gmhA*, which encode the major biofilm determinants of *Y. pestis*, were subjected to the following gene regulation experiments to analyze the detailed regulatory action of CRP on these target genes.

### CRP Has No Regulatory Effect on *hms* Genes at the Transcriptional Level

As revealed by the primer extension and real-time RT-PCR assays, the mRNA levels of each of *hmsH* (Supplementary Figures S1a,b), *hmsT* (Supplementary Figures S2a,b), *hmsC* (Supplementary Figures S3a,b), and *hmsP* (Supplementary Figures S4a,b) were unaltered in Δ*crp* compared with WT. The LacZ fusion assay also revealed that the deletion of *crp* had no effect on the promoter activity of each of *hmsH* (Supplementary Figure S1c), *hmsT* (Supplementary Figure S2c), *hmsC* (Supplementary Figure S3c), and *hmsP* (Supplementary Figure S4c). Moreover, the EMSA assay indicated that His-CRP could not bind to the promoter-proximal region of each of *hmsH* (Supplementary Figure S1d), *hmsT* (Supplementary Figure S2d), *hmsC* (Supplementary Figure S3d), and *hmsP* (Supplementary Figure S4d). Taken together, CRP had no regulatory effect on *hmsHFRS*, *hmsT*, *hmsCDE*, and *hmsP* at the transcriptional level.

### CRP Directly Induces *gmhA* Transcription

**FIGURE 2:**
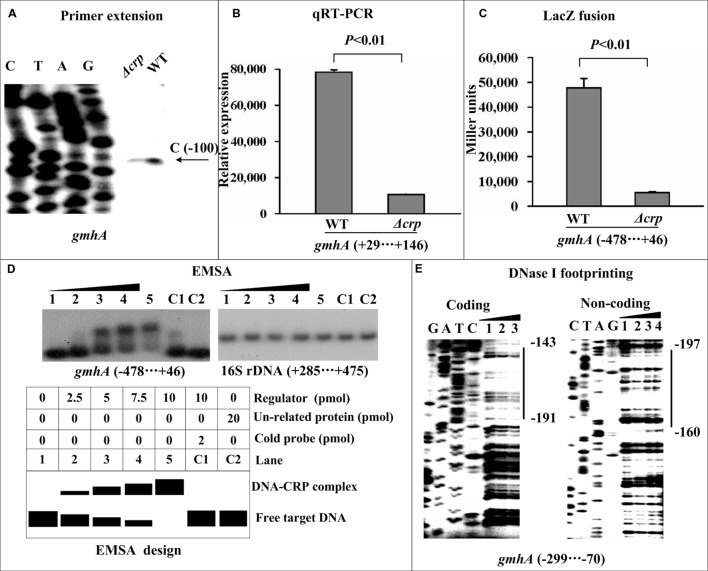
**Direct activation of *gmhA* transcription by CRP**. Lanes C, T, A, and G represent Sanger sequencing reactions. The minus and positive numbers indicated nucleotide positions upstream and downstream of the indicated genes. **(A)** Primer extension. An oligonucleotide primer was designed to be complementary to the RNA transcript of each indicated gene. The primer extension products were analyzed on an 8 M urea-6% acrylamide sequencing gel. The transcriptional start sites are indicated by arrows, showing the nucleotides. **(B)** Quantitative RT-PCR. The mRNA levels of *gmhA* were compared between Δ*crp* and WT. A standard curve was generated for each RNA preparation with the 16S rRNA gene; the relative mRNA level was determined by calculating the threshold cycle (ΔCt) of target genes via the classic ΔCt method. **(C)** LacZ fusion. The target promoter-proximal DNA region was cloned into the *lacZ* transcriptional fusion vector pRW50 and then transformed into the WT or Δ*crp* strain to determine the promoter activity, i.e., the β-galactosidase activity (miller units) in the cellular extracts. **(D)** EMSA. The radioactively labeled promoter-proximal DNA fragments were incubated with increasing amounts of a purified His-CRP protein and then subjected to 4% (w/v) polyacrylamide gel electrophoresis. The band of free DNA disappeared with increasing amounts of His-CRP, resulting in a DNA band with decreased mobility, which presumably represented the DNA-CRP complex. Shown also is a schematic representation of the EMSA design. A DNA fragment from the coding region of the 16S rRNA gene served as a negative control. **(E)** DNase I footprinting. Labeled coding or non-coding DNA probes were incubated with increasing amounts of purified His-CRP and then subjected to a DNase I footprinting assay. The footprint regions are indicated with vertical bars.

The primer extension assay detected a single transcriptional start site for *gmhA*, located 100 bp upstream of *gmhA*, and the mRNA levels of *gmhA* were found to be reduced in Δ*crp* compared with WT (**Figure 2A**), which was confirmed by real-time RT-PCR (**Figure 2B**). Similarly, the LacZ fusion assay showed that deletion of *crp* induced markedly attenuated promoter activity of *gmhA* (**Figure 2C**). The EMSA assay indicated that a purified His-CRP bound to the labeled *gmhA* promoter DNA in a dose-dependent manner. To confirm the specificity of the CRP–DNA association, the EMSA experiments included a partial coding region of the 16S rRNA gene and negative results were obtained (**Figure 2D**). To further locate the precise CRP sites, DNase I footprinting experiments were performed with both coding and non-coding strands of target DNA fragments. The results showed that His-CRP protected a single region within each of the two target DNA fragments tested against DNase I digestion in a dose-dependent manner. The footprint was located from 197 to 143 bp upstream of *gmhA* (**Figure 2E**). Thus, CRP directly promotes the transcription of the *gmhA* gene.

### CRP Indirectly Promotes *waaA* Transcription Through Directly Acting on *phoP*

**FIGURE 3:**
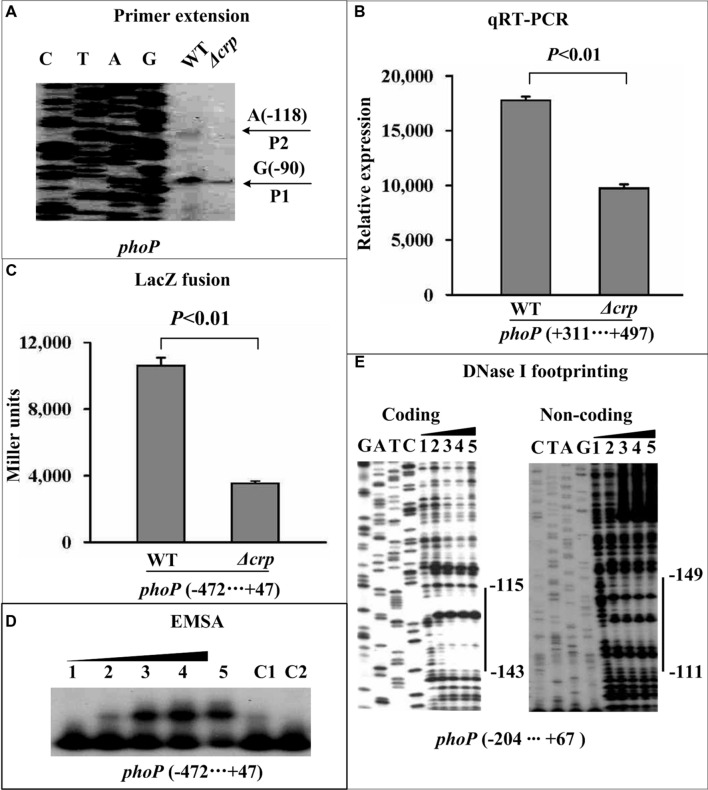
**Direct activation of *phoP* transcription by CRP**. See **Figure 2** for the annotations of primer extension **(A)**, quantitative RT-PCR **(B)**, LacZ fusion **(C)**, EMSA **(D)**, and DNase I footprinting **(E)**.

**FIGURE 4:**
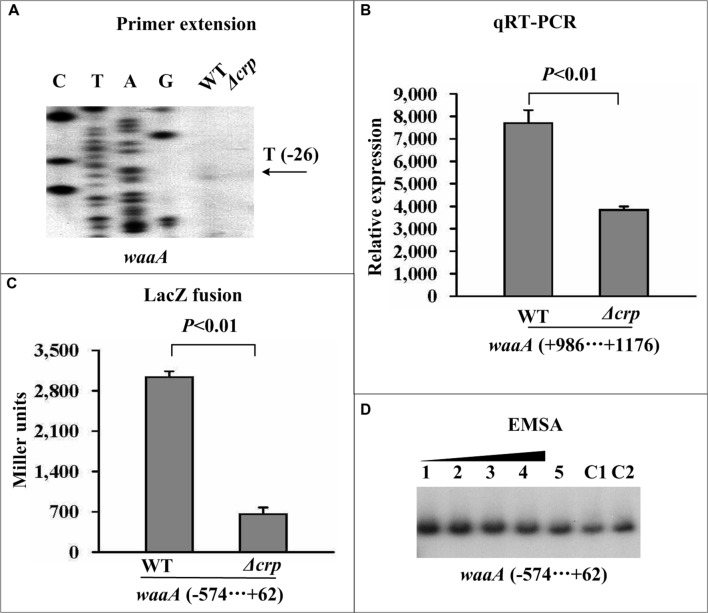
**Indirect activation of *waaA* transcription by CRP**. See **Figure 2** for the annotations of primer extension **(A)**, quantitative RT-PCR **(B)**, LacZ fusion **(C)**, and EMSA **(D)**.

The primer extension and real-time RT-PCR assays revealed that the mRNA levels of *phoP* (**Figures 3A,B**) and *waaA* (**Figures 4A,B**) were dramatically reduced in Δ*crp* compared with WT. Additionally, two distinct transcriptional start sites for *phoP* and a single transcriptional start site for *waaA* were detected by the primer extension experiment. The LacZ fusion assay indicated that the expression of *crp* was essential for the promoter activity of the *phoP* (**Figure 3C**) and *waaA* (**Figure 4C**) genes. The EMSA assay indicated that His-CRP was able to bind in a dose-dependent manner to the promoter-proximal region of the *phoP* gene (**Figure 3D**), but not to that of the *waaA* (**Figure 4D**) gene. The precise CRP-sites for its direct target gene *phoP* were determined by DNase I footprinting experiments, and the results showed that the footprint was located from 149 to 111 bp upstream of *phoP* (**Figure 3E**). Therefore, CRP-dependent activation of *phoPQ*-YPO1632 occurs in a direct manner, while CRP-dependent activation of *waaAE-coaD* occurs in an indirect manner. In addition, our previous study showed that PhoP directly stimulates the transcription of *waaAE-coaD* (Liu et al., 2014), and thereby CRP-mediated activation of *waaAE-coaD* is most likely to function by directly acting on *phoPQ*-YPO1632.

### Promoter Structure of *gmhA*, *phoP*, and *waaA*

**FIGURE 5:**
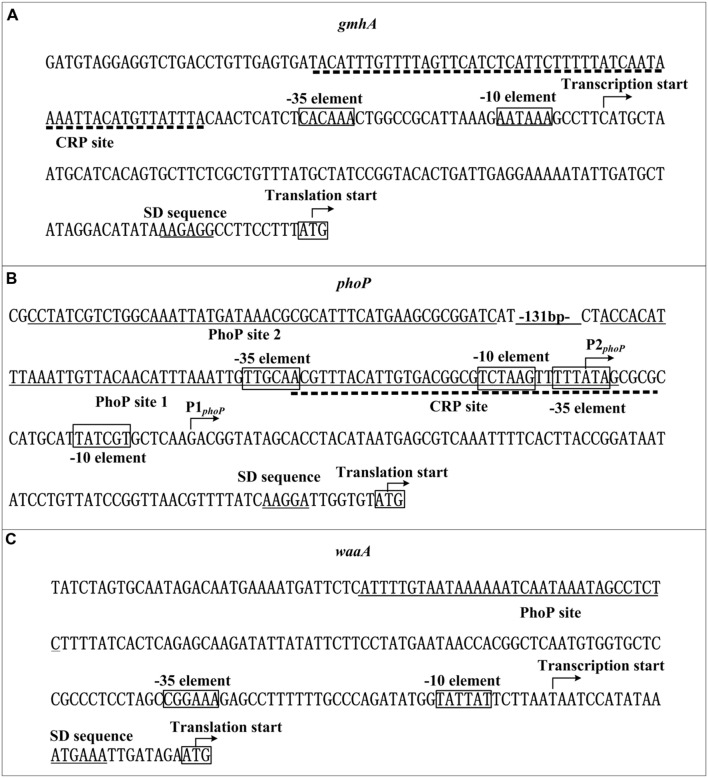
**Organization of promoter-proximal DNA regions**. The promoter-proximal DNA regions of the CRP target genes *gmhA* **(A)**, *phoP* **(B)**, and *waaA* **(C)** are derived from *Y. pestis* CO92. Shown are translation/transcription starts, predicted core promoter –10 and –35 elements, predicted Shine–Dalgarno sequences, CRP and PhoP sites.

The primer extension experiment was used to depict the 5′ termini (transcriptional starts) of RNA transcripts of *gmhA*, *phoP*, and *waaA*, and the –10 and –35 core promoter elements for RNA polymerase recognition were accordingly predicted. The DNase I footprinting experiments were carried out to determine the precise CRP sites for the direct CRP target genes. Collection of data from the translational/transcriptional start sites, the core promoter –10 and –35 elements, the Shine–Dalgarno sequences for ribosomal binding, and the CRP sites (in this study) and PhoP sites (Zhang et al., 2013a; Liu et al., 2014) enabled us to characterize the organization of the promoters of *gmhA* (**Figure 5A**), *phoP* (**Figure 5B**), and *waaA* (**Figure 5C**).

## Discussion

In this study, CRP-dependent activation of biofilm production was observed in *Y. pestis Microtus* strain 201, which confirms the results obtained in *Y. pestis* KIM6+ (a pCD1^-^ derivative of KIM) and CO92 (Willias et al., 2015). Our findings also indicated that CRP-mediated regulation of *Y. pestis* biofilm production has no regulatory relationship with the Hms factors, which is in agreement with the results showing that the deletion of *crp* had no effect on the biosynthesis of c-di-GMP. However, CRP is able to stimulate biofilm development by directly activating the transcription of *gmhA* and meanwhile indirectly promoting the transcription of *waaAE-coaD.* CRP stimulates the transcription of *waaAE-coaD* through acting on *PhoPQ*, a direct activator of *waaAE-coaD* (Liu et al., 2014).

Each of GmhA, WaaA, and WaaE plays a key role in biofilm EPS biosynthesis or modification and is required for biofilm production in *Y. pestis*, but the exact mechanisms of action remain to be determined. Biofilm EPS synthesized in *Y. pestis* cells must be exported through the outer membrane that is predominantly composed of LPS. GmhA, WaaA, and WaaE are all crucial determinants for the biosynthesis of integral LPS. Deletion of each of *gmhA*, *waaA*, and *waaE* results in the formation of an incomplete LPS and, thereby, hinders the export of biofilm EPS, which ultimately leads to a dramatic reduction in biofilm EPS. Given the fact that CRP is a master regulator of the metabolism of alternate carbon sources, it is supposed that CRP-dependent activation of *gmhA* and *waaAE-coaD* enhances *Y. pestis* biofilm production by influencing the carbon source metabolic pathways of the biosynthesis, modification and transportation of biofilm EPS.

The two-component regulatory system PhoP/PhoQ senses and responds to the host environment stimuli of low Mg^2+^, low pH, and antimicrobial peptides, and plays an important role in the flea gut by stimulating the production of flea-borne infectious *Y. pestis* biofilms. CRP senses and responds to the switch in carbon sources, and acts as an activator of *Y. pestis* biofilm formation *in vitro* and on nematodes. Nevertheless, both CRP (this study) and PhoP (Rebeil et al., 2013) have no regulatory effect on the *hms* gene transcription, while the expression of *gmhA* and *waaAE-coaD* are positively regulated by CRP, and concurrently *waaAE-coaD* is directly activated by PhoP. Therefore, CRP- and PhoP-mediated regulation of *Y. pestis* biofilm production may be dependent on a similar mechanism. According to the conclusions obtained in this study and previous studies (Rebeil et al., 2013; Zhang et al., 2013a, 2015; Liu et al., 2014), the complex regulatory interactions of cAMP-CRP and PhoP/PhoQ facilitate the cellular pathways governed by these two regulatory systems to merge into a single global regulatory circuit in *Y. pestis*, which responds to environmental stimuli and controls the expression of genes involved in various functions in flea vectors, thereby enabling better survival and transmission of this pathogen.

The CRP site for *gmhA* is upstream of the promoter –35 element, and thereby CRP-mediated regulation of the *gmhA*promoter may be a class I transcriptional stimulation that depends on the RNA polymerase α subunit C-terminal domain for function (Ishihama, 2000), and PhoP-mediated regulation of *waaA* may operate via a similar regulatory mechanism. However, PhoP recognizes two distinct sites (site 1 and 2) within the *phoP* upstream region, and site 1 overlaps the –10 and –35 regions for P2*_phoP_* and the –35 region for P1*_phoP_*. This may induce the formation of a loop in the *phoP* upstream DNA region that blocks the entry of the RNA polymerase and thereby leads to the autorepression of *phoP*. However, the CRP site for *phoP* partially overlaps with the PhoP site 1, and the interaction of these two regulators may lead to loop relief that facilitates the entry of the RNA polymerase, and thereby stimulates the transcription of *phoP.*

## Author Contributions

DZ and RY conceived the study and designed experimental procedures; LL, HF, HY, YZ, YH performed the experiments and carried out data analysis; DZ, LL, and RY wrote the paper.

## Conflict of Interest Statement

The authors declare that the research was conducted in the absence of any commercial or financial relationships that could be construed as a potential conflict of interest.

## Supplementary material

Figure S1 **CRP had no regulatory effect on *hmsH* at the transcriptional level**. See **Figure 2** for the annotations of primer extension **(a)**, quantitative RT-PCR **(b)**, LacZ fusion **(c)**, and EMSA **(d)**.

Figure S2 **CRP had no regulatory effect on *hmsT* at the transcriptional level**. See **Figure 2** for the annotations of primer extension **(a)**, quantitative RT-PCR **(b)**, LacZ fusion **(c)**, and EMSA **(d)**.

Figure S3 **CRP had no regulatory effect on *hmsC* at the transcriptional level**. See **Figure 2** for the annotations of primer extension **(a)**, quantitative RT-PCR **(b)**, LacZ fusion **(c)**, and EMSA **(d)**.

Figure S4 **CRP had no regulatory effect on *hmsP* at the transcriptional level**. See **Figure 2** for the annotations of primer extension **(a)**, quantitative RT-PCR **(b)**, LacZ fusion **(c)**, and EMSA **(d)**.
