# Supplementary figures and images for: CRP Is an Activator of Yersinia pestis Biofilm Formation that Operates via a Mechanism Involving gmhA and waaAE-coaD
Source: Front Microbiol. 2016 Mar 8;7:295. doi: 10.3389/fmicb.2016.00295 (PMC4782182; doi:10.3389/fmicb.2016.00295)

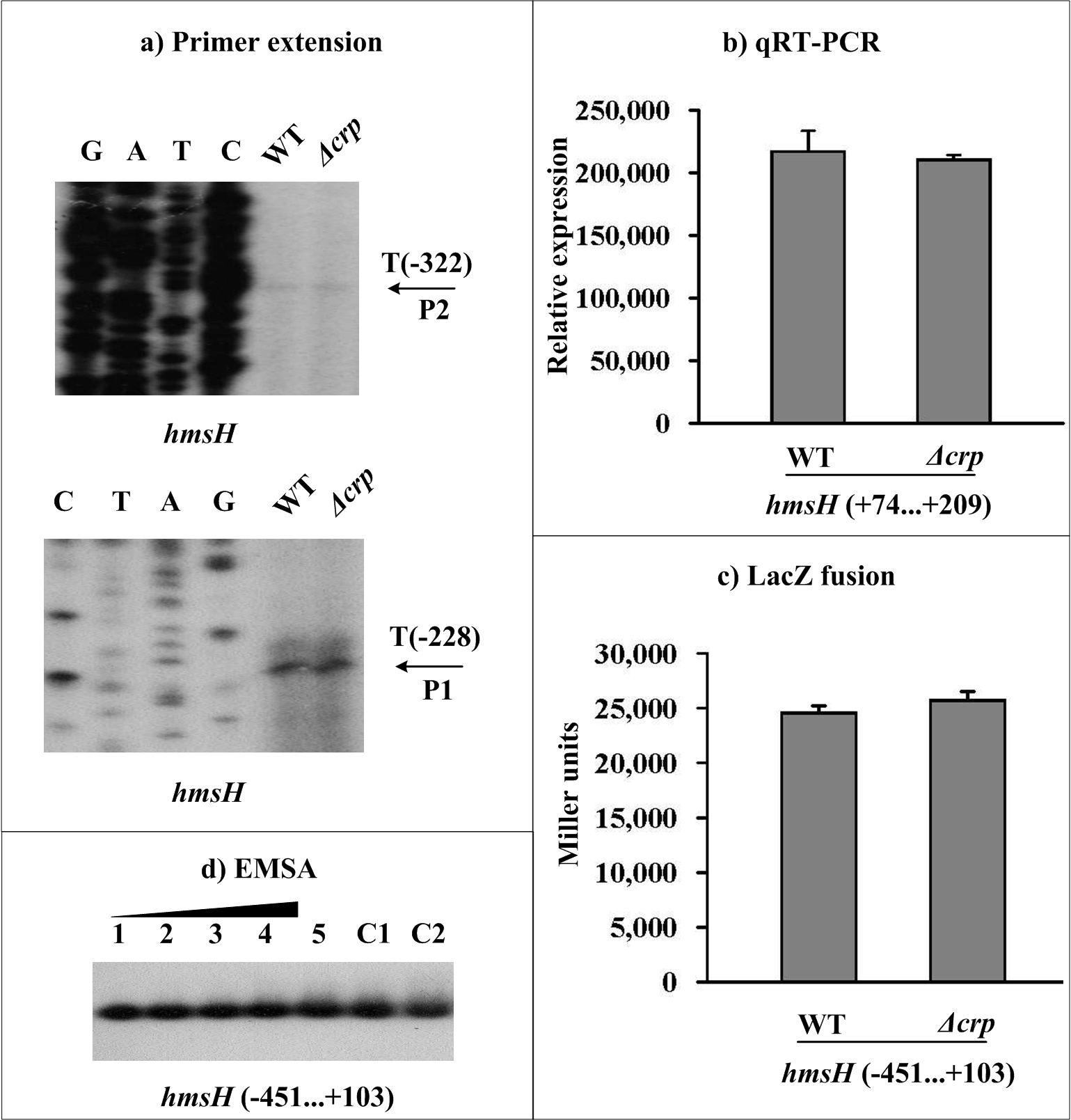

Supplement: Figure S1 — CRP had no regulatory effect on hmsH at the transcriptional level. See Figure 2 for the annotations of primer extension (a), quantitative RT-PCR (b), LacZ fusion (c), and EMSA (d). [file Image_1.JPEG]

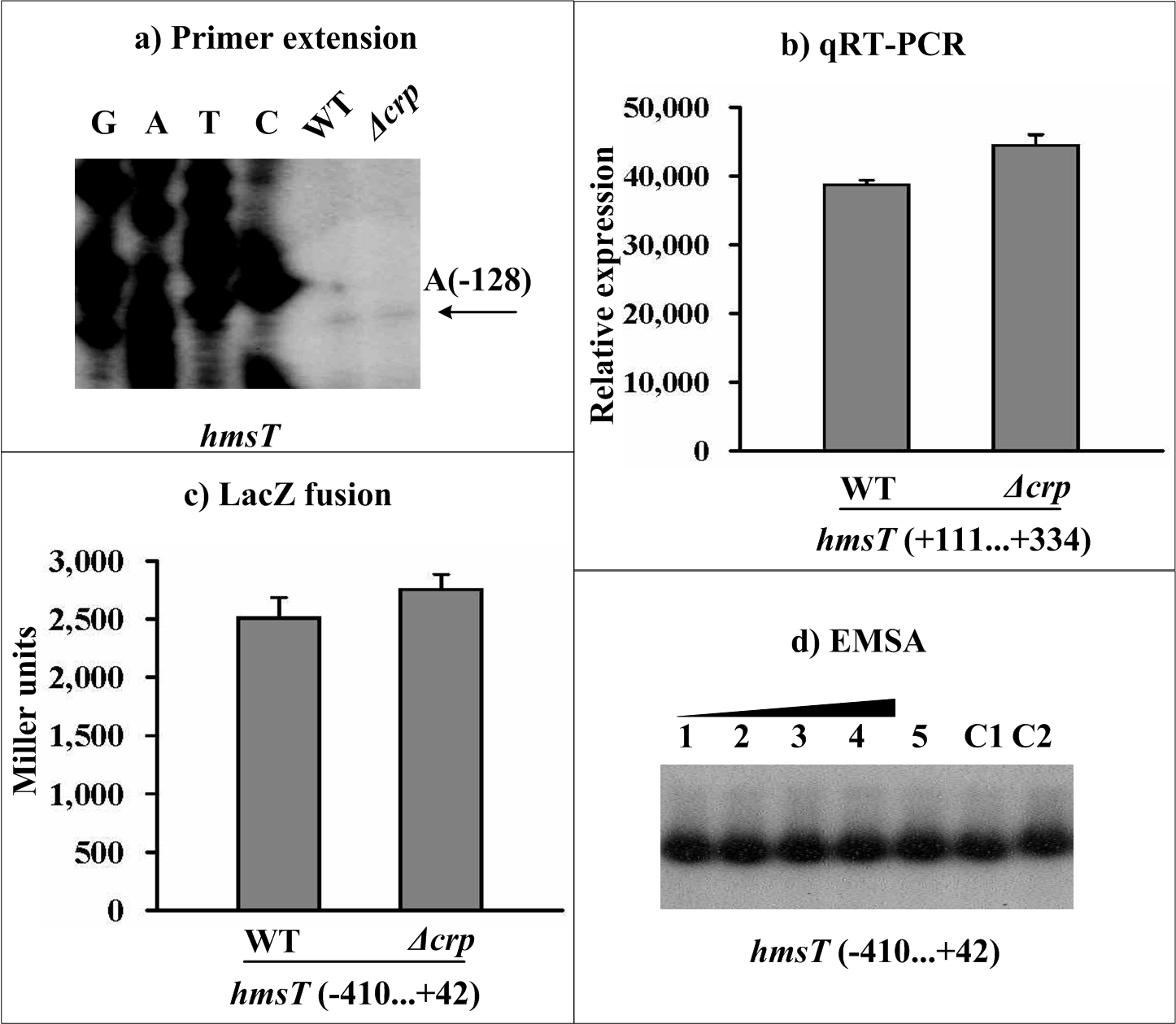

Supplement: Figure S2 — CRP had no regulatory effect on hmsT at the transcriptional level. See Figure 2 for the annotations of primer extension (a), quantitative RT-PCR (b), LacZ fusion (c), and EMSA (d). [file Image_2.JPEG]

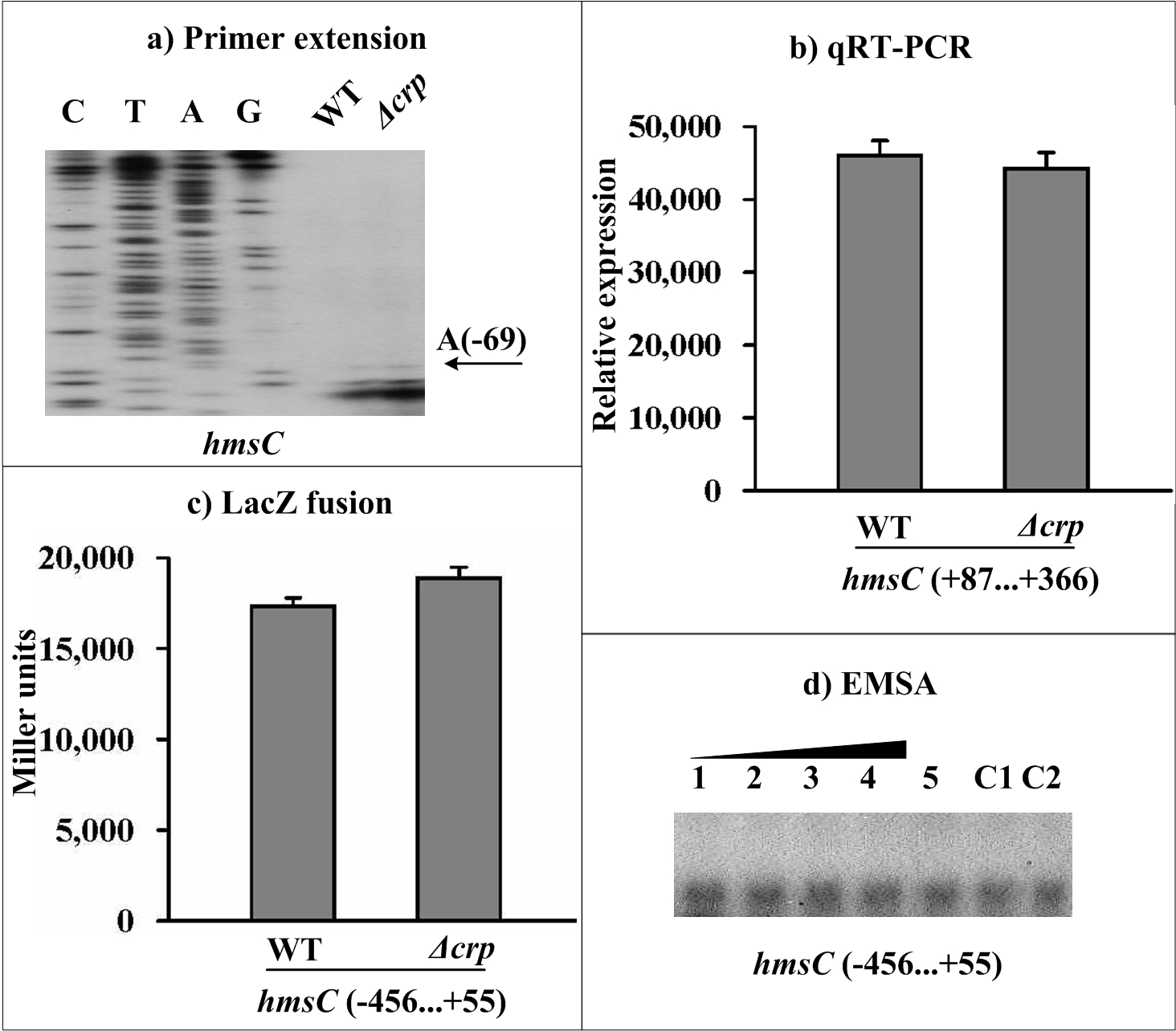

Supplement: Figure S3 — CRP had no regulatory effect on hmsC at the transcriptional level. See Figure 2 for the annotations of primer extension (a), quantitative RT-PCR (b), LacZ fusion (c), and EMSA (d). [file Image_3.JPEG]

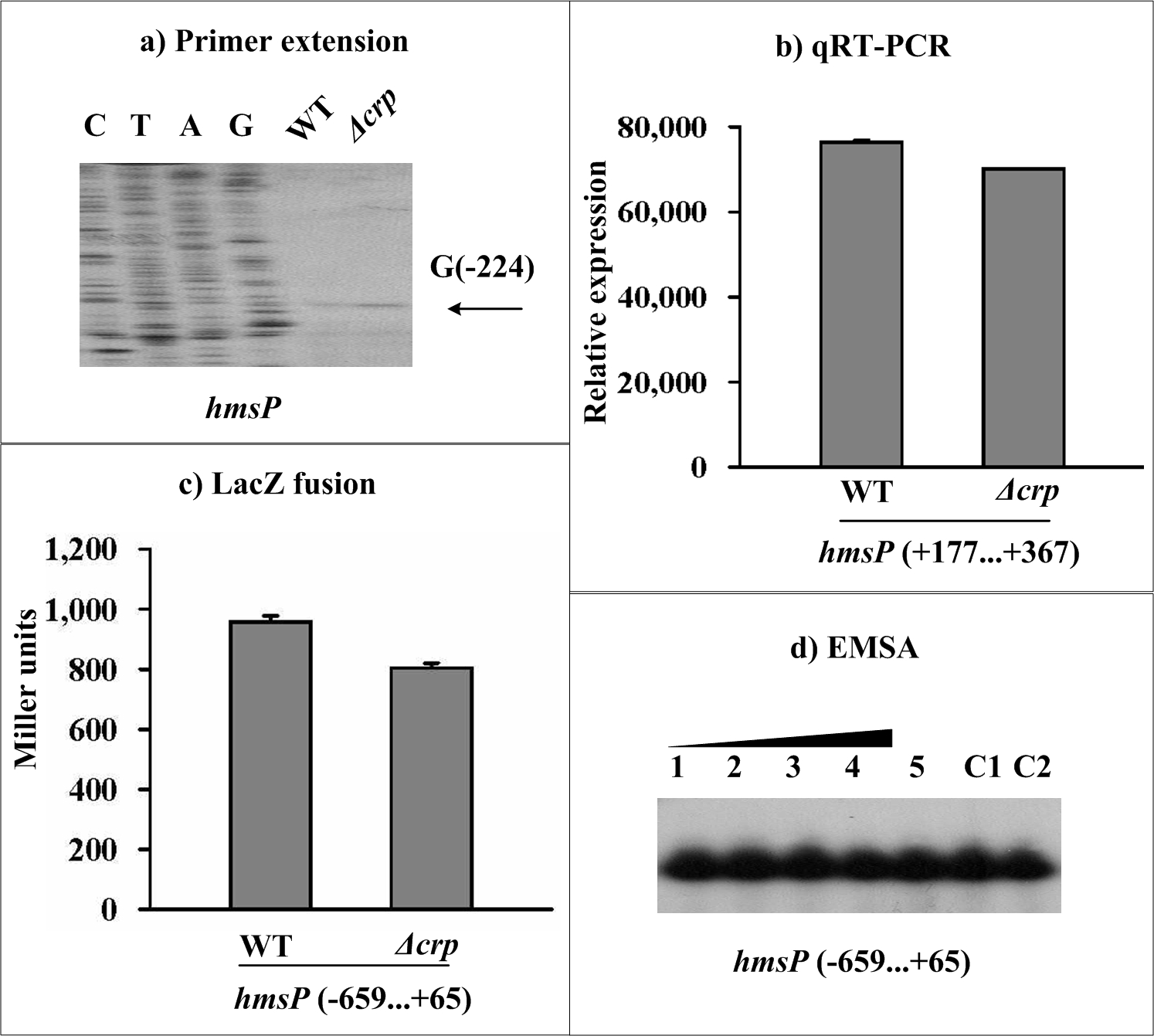

Supplement: Figure S4 — CRP had no regulatory effect on hmsP at the transcriptional level. See Figure 2 for the annotations of primer extension (a), quantitative RT-PCR (b), LacZ fusion (c), and EMSA (d). [file Image_4.JPEG]
