# Supplementary material for: CRP Is an Activator of Yersinia pestis Biofilm Formation that Operates via a Mechanism Involving gmhA and waaAE-coaD
Source: Front Microbiol. 2016 Mar 8;7:295. doi: 10.3389/fmicb.2016.00295 (PMC4782182; doi:10.3389/fmicb.2016.00295)
Supplement: Supplementary file 5 [file Table_1.DOCX]

**Table S1 Primers used in this study**

| **Target**  **gene** | **Primers (5'-3'; F/R)** |
| --- | --- |
| **Primer extension** | |
| *gmhA* | /GGAAGGCCTCTTTATATGTCC |
| *phoP* | /ACCCGCATACACCAATCCTT |
| *waaA* | /GTAAACGCAGCCAAATCAGAG |
| *hmsH* | /TATTGTTGCAAAGTCATTATAGGAT |
| *hmsT* | /GGTATTTATTCCGACATCACGAC |
| *hmsC* | /AGTAGCGGTAGTCATTTTTACG |
| *hmsP* | /CCATCGAGTAAGTTGTGATCC |
| **Quantitative RT-PCR** | |
| *gmhA* | TGAACGAAGCAGCAGACA/ GACAATACTTTGCCACCC |
| *phoP* | ATCTGGAAGAGGTCATTGC/CTGCGTTGCGGATAAGG |
| *waaA* | TTGAACGTGGCGGTCATAAC/GAGGCGGCAATCTTCATCAG |
| *hmsH* | CTGGCTTTGTCGTTAATCCTG/TGCCCACTCTGCAATGGAC |
| *hmsT* | CAGTATGCTATCATCGTCGC/GTAGACCGATGAGGATTG |
| *hmsC* | GGGCGTTTATCTATTCTTAC/GAGTGAGTTATTGGGAAGTG |
| *hmsP* | GGTAAGGCGCTCATTAACGA/TATCAACGCTGAGTATGGCC |
| **LacZ reporter fusion** | |
| *gmhA* | GCGGGATCCATGACGCTACGGAAAATC/  GCGAAGCTTTGTCTGCTGCTTCGTTCA |
| *phoP* | CCGGAATTCTGATGCCAGCAAAGACG/  CGCGGATCCAGATGGTGACGCAACAAC |
| *waaA* | GCGGGATCCTGGATCGCCCAAACATTACG/  GCGAAGCTTAAACGCAGCCAAATCAGAGG |
| *hmsH* | GCGGGATCCACTTTGCTGAAGACTTGTCACG/ GCGAAGCTTCCGCCATAGCAGGATTAACG |
| *hmsT* | GCGGAATTCGCCCAGTACAGGTAACAAGG/ GCGGGATCCCTGATCGTAGGAGTGGCTATTC |
| *hmsC* | TCTGGATCCCTTACTGGTTGCTATTGCC/ TCTAAGCTTGAGGTTCATGATGTTCATCA |
| *hmsP* | GCGGGATCCAGCGATGGTAGAAGTGAATCAG/ GCGAAGCTTTTGCGATACTCTAATGGAAGGC |
| **EMSA** | |
| *gmhA* | GCGGGATCCATGACGCTACGGAAAATC/  GCGAAGCTTTGTCTGCTGCTTCGTTCA |
| *phoP* | CCGGAATTCTGATGCCAGCAAAGACG/  CGCGGATCCAGATGGTGACGCAACAAC |
| *waaA* | GCGGGATCCTGGATCGCCCAAACATTACG/  GCGAAGCTTAAACGCAGCCAAATCAGAGG |
| *hmsH* | GCGGGATCCACTTTGCTGAAGACTTGTCACG/ GCGAAGCTTCCGCCATAGCAGGATTAACG |
| *hmsT* | GCGGAATTCGCCCAGTACAGGTAACAAGG/ GCGGGATCCCTGATCGTAGGAGTGGCTATTC |
| *hmsC* | TCTGGATCCCTTACTGGTTGCTATTGCC/ TCTAAGCTTGAGGTTCATGATGTTCATCA |
| *hmsP* | GCGGGATCCAGCGATGGTAGAAGTGAATCAG/ GCGAAGCTTTTGCGATACTCTAATGGAAGGC |
| **DNase I footprinting** | |
| *gmhA* | CCGGTGATAGAACAACACAC/ ACAGCGAGAAGCACTGTGATG |
| *phoP* | GGCGAGTCAGTGACCAAGCG/  CGCTCATTATGTAGGTGC |
